# Epidemiology of Cow’s Milk Allergy

**DOI:** 10.3390/nu11051051

**Published:** 2019-05-10

**Authors:** Julie D. Flom, Scott H. Sicherer

**Affiliations:** Elliot and Roslyn Jaffe Food Allergy Institute, Division of Allergy, Department of Pediatrics, Kravis Children’s Hospital, Icahn School of Medicine at Mount Sinai, New York, NY 10029, USA; scott.sicherer@exchange.mssm.edu

**Keywords:** cow’s milk allergy, epidemiology, natural history, prevalence

## Abstract

Immunoglobulin E (IgE)-mediated cow’s milk allergy (CMA) is one of the most common food allergies in infants and young children. CMA can result in anaphylactic reactions, and has long term implications on growth and nutrition. There are several studies in diverse populations assessing the epidemiology of CMA. However, assessment is complicated by the presence of other immune-mediated reactions to cow’s milk. These include non-IgE and mixed (IgE and non-IgE) reactions and common non-immune mediated reactions, such as lactose intolerance. Estimates of prevalence and population-level patterns are further complicated by the natural history of CMA (given its relatively high rate of resolution) and variation in phenotype (with a large proportion of patients able to tolerate baked cow’s milk). Prevalence, natural history, demographic patterns, and long-term outcomes of CMA have been explored in several disparate populations over the past 30 to 40 years, with differences seen based on the method of outcome assessment, study population, time period, and geographic region. The primary aim of this review is to describe the epidemiology of CMA. The review also briefly discusses topics related to prevalence studies and specific implications of CMA, including severity, natural course, nutritional impact, and risk factors.

## 1. Introduction

Cow’s milk allergy (CMA) is defined as an immune-mediated response to proteins in cow’s milk that occurs consistently with ingestion. It is one of the most common food allergies in early life [1,2,3] with an estimated prevalence in developed countries ranging from 0.5% to 3% at age 1 year (reviewed in [1,4,5,6,7,8]). In this review, we summarize prevalence estimates of cow’s milk allergy worldwide and discuss the clinical and public health implications of understanding risk factors for development and persistence of CMA. Diagnosis, prevention, and treatment are briefly discussed in relation to their implications on prevalence estimates.

## 2. Subtypes of Immune-Mediated Reactions to Cow’s Milk

CMA refers to immune-mediated reactions to cow’s milk that are categorized as immunoglobulin E (IgE)-mediated, non-IgE mediated, and mixed (IgE combined with non-IgE) [4,8,9,10,11,12,13]. This review focuses on IgE-mediated cow’s milk allergy (CMA), a type I hypersensitivity reaction in which symptoms usually occur within minutes to 1 to 2 hours of ingestion. IgE antibodies to proteins in cow’s milk bind to mast cells, and subsequent exposure to the protein leads to mast cell degranulation and release of mediators, including histamine. This leads to symptoms including urticaria; angioedema; throat tightness; respiratory symptoms, including difficulty breathing, coughing, and wheezing; gastrointestinal symptoms, including abdominal pain, vomiting, and diarrhea; and cardiovascular symptoms, including dizziness, confusion, and hypotension [8,9,11]. Approximately 60% of those with CMA have the IgE-mediated form, although estimates vary by study population and age [8,12,14].

Mixed and non-IgE mediated forms of CMA have different underlying mechanisms, presentation, and implications, which complicate assessment of the epidemiology of IgE-mediated CMA (Table 1). Mixed forms of CMA (both IgE and non-IgE mediated) include atopic dermatitis, allergic eosinophilic esophagitis, and eosinophilic gastritis. Given the lack of validated testing, cow’s milk as a trigger for these diseases is often identified through history and elimination diets. Non-IgE mediated forms of CMA include cow’s milk enteropathy [12], food protein induced proctitis/proctocolitis [6], food protein induced enterocolitis syndrome (FPIES) [13], and Heiner syndrome [8]. FPIES generally manifests with severe vomiting at least 2 hours after ingestion, with negative skin and blood testing (reviewed in [4]) and no validated diagnostic testing [6]. One large Israeli study estimated cow’s milk FPIES prevalence in infancy at 0.34% [15]. Non-immune mediated reactions, such as lactose intolerance, typically lead to overestimates of prevalence in population-based studies that rely on self-report [8,10,14,16].

## 3. Diagnosis of CMA

The remainder of this review focuses on IgE-mediated CMA. Methods available for diagnosis have limitations, which impact the ability to elucidate the underlying epidemiology (Table 1) [3,9,17,18]. The gold standard for diagnosis is the double blind, placebo-controlled oral food challenge (DBPCFC) [18]. The unblinded oral food challenge (OFC) is less rigorous, but well validated, especially in young children [19]. However, both tests are time- and resource-intensive and carry inherent risk of anaphylaxis. OFCs are therefore not always appropriate for use in clinical practice or especially in large epidemiologic studies. Studies that employ these methods may have incomplete assessments due to parental refusal and safety concerns in highly atopic children [20]. Objective measures used routinely in both epidemiologic studies and clinical practice include serum-specific IgE (sIgE) and skin prick tests (SPT). These two tests predict the likelihood of reaction, but, in isolation, are not sufficient for diagnosis (reviewed in [9]). Sensitization measured via SPT is often defined as a wheal at least 3 mm larger than the negative control. Cow’s milk sIgE, measured by in vitro immunoassay, measures IgE binding to specific proteins; sensitization is defined as detectable sIgE (often sIgE ≥ 0.35 kU/L, sometimes ≥ 0.10 kU/L). Self-report of CMA [16] and reliance on sensitization based solely on serum sIgE and/or skin testing as a means to identify CMA tend to overestimate prevalence [18,21,22]. Further, variations in assays for sIgE can lead to conflicting interpretations and limit comparability between study populations [23].

Prevalence estimates are also impacted by variation in tolerance as defined by the types of milk products that are ingested. Approximately 70% of patients with IgE-mediated cow’s milk allergy who would react to whole cow’s milk products (e.g., milk, ice cream, yogurt) tolerate extensively heated, baked milk products (e.g., cookie, muffin) [24] because baking alters certain proteins in milk, leading to a loss of conformational epitopes and decreased allergenicity (reviewed in [6,25,26]). This further complicates our understanding of the epidemiology and natural history and it is rare that epidemiologic studies distinguish tolerance of baked cow’s milk.

## 4. Prevalence of CMA

### 4.1. Prevalence of IgE-Mediated CMA: Meta-Analysis and Systematic Reviews

Despite the limitations in assessment, there are a large number of studies in the US and worldwide that attempt to estimate the incidence or prevalence of CMA. There are select meta-analyses and systematic reviews that summarize existing data on CMA in the US and worldwide [2]; however, there is heterogeneity between the studies, which complicates comparisons and summary estimates (Table 2A). Rona et al. [3] performed a meta-analysis of papers on food allergy published from January 1990 to December 2005. They reported a range in prevalence by study methodology, with estimates from studies relying on self-report ranging from 1.2% to 17%, those using SPT alone from 0.2% to 2.5% and sIgE alone from 2% to 9%, studies using symptoms and sensitization (SPT ≥ 3 mm or sIgE ≥ 0.35) ranging from 0% to 2.0%, and those relying on food challenges (OFC or DBPCFC) ranging from 0% to 3.0%.

Nwaru et al. performed a systematic review and meta-analysis of CMA prevalence in European studies published between 2000 and 2012 and included 42 primary articles on CMA [27]. They also observed a variation in prevalence by means of diagnosis. By self-report, the point prevalence of CMA was 2.3% (95% CI 2.1–2.5); by SPT alone, 0.3% (95% CI 0.03–0.6); and by sIgE alone, 4.7% (95% CI 4.2–5.1). The prevalence of CMA diagnosed by food challenge was 0.6% (95% CI 0.5–0.8) and by food challenge or reported history of CMA was 1.6% (95% CI 1.2–1.9). The authors also reported a higher prevalence among younger ages.

These studies demonstrate that appreciable variation is seen in estimates varying by factors, including geographic region, source population (high risk referral vs. general population), age and participation rates, and limitations of diagnosis [9,18].

### 4.2. Prevalence of IgE-Mediated CMA: Select Studies

Systematic reviews and meta-analyses are helpful in characterizing overall trends and observing differences in prevalence estimates. However, there are nuances in individual studies that provide a number of insights into the epidemiology of CMA as demonstrated by the studies reviewed below (Table 2B). 

The EuroPrevall birth cohort included 12,000 children from nine countries in Europe enrolled at birth and followed through the age of 24 to 30 months [28]. Diagnosis was based on DBPCFC administered to all infants with no regular consumption of cow’s milk and evidence of sensitization or a reported history of reaction or improvement in symptoms after dietary elimination. They observed an overall prevalence of CMA of 0.59% (adjusted for loss to follow-up and DBPCFC placebo reactors), with prevalence ranging from 0% to 1.3% in various countries. The strengths of the study include the large sample size, a source population representative of the general population, and use of DBPCFC for all participants with possible allergy at age 1 year. The study also carefully distinguished between IgE and non-IgE mediated CMA. However, it is limited to European countries and it is not clear if the results can be generalized.

In Israel, Katz et al. performed a prospective study of all births at a single medical center over 2 years (*n* = 13,234) and identified a cumulative incidence of 0.5% with a mean age of onset of 3.9 months [29]. The study was strengthened by its prospective design, high recruitment rate (98.4%), and use of OFC for almost 75% of diagnoses (with the exception of those with a history of life-threatening reaction or family refusal). In an unselected population of 3 year olds in Denmark (started in late 1990s), Osterballe et al. reported a CMA prevalence of 0.6% [30]. In a birth cohort of 1749 infants in Denmark, Host et al. observed an incidence of CMA in the first year of life of 2.2% (95% CI 1.5%–2.9%); 0.5% (95% CI 0.2%–0.9%) were confirmed by OFC [31].

The studies described above benefit from recruitment in early life and some have prospective follow-up enabling estimates of incidence. Additional studies, primarily in the US, provide population-based estimates, which are also important for informing public health. Liu et al. [32] used data from the National Health and Nutrition Examination Survey (NHANES) 2005–2006 to estimate the prevalence of food allergy to select foods. They measured cow’s milk sIgE from stored serum samples from 8203 child and adult participants. The prevalence of milk sensitization (defined as sIgE ≥ 0.35) was 5.7% overall and the estimated clinical prevalence of CMA (based on a 95% positive predictive value for milk of 15 kU/L) was 0.40. The prevalence was appreciably higher among younger children (aged 1 to 5 years), with 22% sensitized and an estimated clinical food allergy rate of 1.8%. Among all other ages (aged 6 to >60 years), estimated rates ranged from 0.16% to 0.49%. This study is limited by the lack of data on clinical reactivity; however, the authors made use of a large, nationally representative sample and prevalence estimates are based upon a conservative estimate of sIgE. Gupta et al. [7] performed a cross-sectional study in a representative sample of 38,380 children from the US population using a telephone survey. Overall prevalence of CMA was 1.7% (95% CI 1.5–1.8) and peaked in children aged 0 to 5 years at 2.0%, with a decrease in prevalence over childhood that did not reach statistical significance.

There are fewer reports of the prevalence of CMA in adults. Based on what is currently understood about the natural history of CMA, prevalence in adults is expected to be lower and estimates are often around 0.5% [3]. However, higher estimates have been reported, with some misclassification likely due to inaccuracies in self-report. McGowan et al. [33] investigated the self-reported prevalence of CMA in the National Health and Nutrition Examination Survey (NHANES) 2007–2010 and, surprisingly, report a higher prevalence in adults (2.64%) than children (1.94%), with an overall sample average of 2.47%. When the authors excluded those with consumption in the previous month, the prevalence of CMA in the overall sample decreased to 1.62% [33].

Recent data from the US also reports a higher prevalence of CMA in adults. In a representative cross-sectional survey of 40,443 adults who completed an internet or telephone-based survey in 2015–2016, Gupta et al. [34] observed a prevalence of 1.9% (95% CI 1.8%–2.1%), which peaked at the ages of 18 to 29 years at 2.4%. Diagnosis of CMA was not based on standardized assessment across the population, with the authors using objective criteria to identify “convincing” allergy, defined as those with a history of reaction limited to a symptom list defined by an expert panel. They further used physician diagnosis and history of severe reactions to limit misclassification. Of those with CMA in adulthood, 47.1% (95% CI 43.0%,51.3%) were physician diagnosed and 39.3% had severe reactions (95% CI 35.2%,43.5%). If these estimates are accurate, they would support an increase in prevalence in adults. Interestingly, 22.7% (19.6%,26.3%) reported onset in adulthood. There is likely some misclassification by self-report, and if replicated or validated, further assessment of the underlying mechanisms of adult-onset cases would be warranted. 

### 4.3. Patterns of CMA Prevalence Over Time

Several lines of evidence suggest an increase in sensitization and reported FA over time [9,35]; however, it is unclear if there has been a change in the prevalence of CMA. Challenges in estimating patterns of CMA over time are complicated by its natural history of resolution in a majority of individuals, as well as non-IgE mediated CMA, non-immune mediated reactions, and heterogeneity within CMA, with a subset able to tolerate baked cow’s milk or having a high threshold of ingestion of unheated cow’s milk prior to having symptoms. If an increase in prevalence is not due to increased self-report, it could be due to changes in sensitization, incidence, or persistence of CMA.

Studies demonstrate a relative stability of estimates of sensitization over time. In a population-based study in the US, McGowan et al. [5] compared the prevalence of sensitization to cow’s milk using stored serum samples in children aged 6 to 19 years in NHANES III (1988–1994, *n* = 4995) and NHANES 2005–2006 (*n* = 2901). Sensitization was based on serum cow’s milk specific IgE (≥0.35) and was remarkably similar over time with a sensitization of 8.3% in 1988–1994 and 8.1% in 2005–2006. There were no significant differences observed using alternate cutoffs for moderate- and high-level sensitization [36]. The study was limited to older children (there was no stored serum for younger NHANES III participants), but demonstrates fairly constant levels of sensitization over approximately 15 years.

Peters et al. also assessed changes in sensitization over time [37] in two high risk cohorts in Australia (high risk defined as at least one first degree relative with atopy) in the Melbourne Atopy Cohort Study (MACS) (born 1990–1994) and the high-risk subgroup of the HealthNuts study (born 2006–2010). In both studies, children had SPT to milk and other allergens at 12 months and the prevalence of sensitization was similar at 2.4% and 2.6%, respectively. These studies were careful to use similar definitions of CMA, which strengthens internal comparisons, although a high-risk population may not be representative of general population trends.

Prevalence may also vary over time if there are changes in the rate of resolution. There is some evidence that the rate of resolution is slowing for some foods, including milk, leading to resolution at older ages [9,38]. This is supported by the reports of higher than expected rates of CMA in US adults described above [34]. However, there are inherent methodologic challenges in comparing resolution over time between different studies for several reasons, including heterogeneity in the source population and the method of follow up.

## 5. Natural History of CMA

The natural history of CMA is unique in that resolution is common. However, this complicates prevalence estimates. CMA most frequently presents in infancy and early childhood, typically in the first 12 months of life, and tends to resolve with age (reviewed in [1,4,8,11,19]). For reasons previously described, there is heterogeneity in the estimated rates of resolution. Lack of recognition of resolution can lead to unnecessary exclusion of cow’s milk with subsequent nutritional and growth implications. In a study in Swedish schoolchildren aged 12 years, of those with allergy to milk reporting complete avoidance (*n* = 87), only 3% had true IgE-mediated CMA on DBPCFC and 32% (*N* = 28) had resolved CMA [14]. This demonstrates the need to consider follow-up evaluations both for population patterns and to address management on an individual patient level.

Several studies of natural history have been conducted in high risk cohorts, which are better powered to identify predictors of resolution. In the Consortium for Food Allergy Research (CoFAR) [39], 512 individuals aged 3 to 15 months selected for 1) moderate-to-severe atopic dermatitis (AD) and positive SPT to egg or milk, or 2) clinical history of egg or milk allergy with confirmatory SPT were followed. At a mean age of 53 months, 53% of participants with CMA developed tolerance (defined using clinical history and OFC if needed). Similarly, in the EuroPrevall population, of those with DBPCFC-confirmed CMA, 57% developed tolerance within 1 year [28]. In the Isle of Wight cohort, CMA prevalence (defined as exposure to cow’s milk with IgE-mediated symptoms within 4 hours) decreased from a maximum of 3.5% at age 1 year to 0.3% at age 18 years [40].

Sensitization has been shown to decline with age. In a high-risk cohort in Australia, there was an 8.7% prevalence of milk sensitization at 12 months, which decreased to <5% at age 18 years [41]. This study was biased by higher rates of loss to follow-up in participants with lower SES, younger parents, and lower rates of food sensitization at age 2 years, limiting the generalizability and possibly overestimating prevalence in older children. In large samples from the general population, it is often more feasible to use self-report; however, this can bias estimates. In a cross-sectional study of US children aged 0 to 18 years, no significant variation in self-reported CMA was observed by age although a non-significant higher prevalence was observed in children under 5 years (2.0%) than in older groups (1.4%–1.6%). The study was limited by a lack of standardized assessment and the cross-sectional study design, which does not provide for prospective follow-up over time [7].

Skripak et al. [42] reviewed the charts of 4117 patients seen at private and university-based practices. Of these, 1073 had a CMA diagnosis and 807 had complete data. Diagnostic criteria for CMA were (1) symptoms with exposure to cow’s milk, (2) improvement in atopic dermatitis or other symptoms with avoidance, or (3) positive SPT or sIgE. They evaluated resolution within approximately 12 months (via home introduction with negative sIgE and no reaction or OFC) and identified rates of resolution at age 4, 8, 12, and 16 years of 19%, 42%, 64%, and 79%, respectively. This rate of resolution may be lower than other studies because the authors included a high risk cohort and may have missed resolved cases given that OFC was restricted to those with at least a 50% chance of passing.

Other factors impacting the determination of natural history include older age at recruitment, which may be associated with lower estimates of resolution [11], and type of CMA. Non-IgE mediated CMA is associated with a faster rate of resolution than IgE-mediated CMA and has different associated risk factors and outcomes [28,31,43,44]. For example, in EuroPrevall, 100% of patients with non-IgE mediated CMA developed tolerance within 1 year (compared with 57% of those with IgE-mediated CMA) [28].

Whereas there is clearly notable heterogeneity among studies to date, in a combined analysis of those with CMA in infancy, by age 5 years, 50% developed tolerance and by early adolescence, 75% developed tolerance [45]. Understanding these overall trends and reasons for variation has important implications for management and treatment.

## 6. Factors Associated with Resolution

There is great interest in understanding factors associated with the development of tolerance because the prediction of resolution may impact decisions about treatment and the timing of diagnostic tests. In general, severe initial reactions and co-morbid atopic conditions are associated with lower rates of resolution [9,46]. Wood et al. [47] investigated predictors of development of tolerance in a subset of milk allergic patients in CoFAR and reported a significantly increased rate of resolution among those with lower baseline sIgE, SPT size, and less severe eczema [47] (reviewed in [39,48]). Specifically, among those with a baseline SPT < 5 mm, CMA resolved in 72%; among those with baseline SPT 5 to 10 mm, CMA resolved in 52%; and among those with a SPT > 10 mm, 37% developed tolerance. Thus, a baseline SPT < 5 mm was associated with a 3.7-fold higher chance of resolution compared to a baseline SPT > 10 mm. Baseline sIgE levels were similarly helpful in prediction. Among those with the lowest level of sensitization (sIgE < 2), 72% developed tolerance; among those with moderate levels (sIgE 2–10), 54% developed tolerance; and among those with higher levels (sIgE > 10), 23% developed tolerance [47].

The main proteins in cow’s milk include casein (which accounts for approximately 80% of the total milk protein) and whey (which accounts for approximately 20%). The primary whey allergens are α-lactalbumin (Bosd4), β-lactoglobulin (Bosd5) and bovine serum albumin (Bosd6). Extensive heating decreases the allergenicity of whey proteins. Serum sIgE to casein can be particularly useful for both the prediction of resolution and management with regard to offering baked forms of milk. Higher levels of sIgE to casein have been associated with persistent CMA [20,48]. Lower levels of casein sIgE are associated with an increased likelihood of tolerating milk in a baked form. There is evidence that approximately 70% of patients with CMA can tolerate baked milk [25,26] and that exposure to baked milk may lead to faster development of tolerance to all forms of milk [1,49,50,51]. The ability to tolerate baked milk may also be a marker of a transient CMA phenotype with demonstrated immunologic changes, including increased IgG4 and decreased SPT, in patients ingesting baked milk [25]; however, the relative contributions of the phenotype and impact of intervention are still unknown [1,24,49].

## 7. Severity

Cow’s milk is among the most common causes of food-induced anaphylaxis, along with peanuts and tree nuts [4,52]. In a prospective study of 512 children with likely milk or egg allergy, and many having multiple food allergies, including peanut (median 35 months, range 0–48 months), cow’s milk was the most common cause of allergic reactions to food [53]. Cow’s milk has also been implicated as a cause of severe reactions in several studies, including the European Anaphylaxis Registry, in which cow’s milk was in particular an important cause of severe reactions in those less than 6 years old [54]. In another population, in 495 reactions to cow’s milk, 9.1% were classified as severe [53]. Fatality is rare, but in a case series of fatalities in a European population with data on 1970 children in 10 countries with anaphylaxis to food, there were a total of 5 fatal anaphylactic reactions and 2 were attributed to cow’s milk [54].

## 8. Nutritional and Growth Concerns

In addition to risk of severe and life-threatening anaphylaxis, CMA has important nutritional implications with impacts on growth that persist through adulthood [55]. Sinai et al. compared adult height in 87 patients with CMA compared with 36 individuals with no dietary limitations and found that patients with lifelong CMA had an average 3.8 cm lower adult height than controls [56]. The study was strengthened by the consideration of potential confounders, including chronic steroid exposure in the setting of asthma and use of attained height. Patients with CMA also have higher rates of vitamin D deficiency (reviewed in [57]).

## 9. Risk Factors for CMA

Risk factors and prevention are discussed briefly here in relation to epidemiology. Among children, those with CMA are more likely to be male (up to 2-fold higher risk; this reverses in adulthood with 80% of those with CMA being female). Those with CMA are also more likely to have other atopic diseases, with the prevalence of multiple food allergies identified in over 90% of CMA patients in a high risk population [29,42] and high rates of asthma, atopic dermatitis, and allergic rhinitis [31]. There is some evidence of variation by race/ethnicity, with studies suggesting non-Hispanic black and non-Hispanic white children are more likely to be sensitized to milk, based on serum IgE [32,58]. McGowan et al. compared the prevalence of sensitization by race in NHANES participants from 1991 to 1994 (aged 6 to 19 years) using sIgE to cow’s milk measured in stored samples and found statistically significant differences, with the lowest rates of sensitization in white participants compared to black and Mexican-American participants (prevalence in non-Hispanic white, non-Hispanic black, and Mexican-American participants was 5.6%, 12.8%, and 12.2%, respectively) [59]. When estimates from NHANES III and 2005–2006 were compared, there were no changes in the prevalence of sensitization among any race/ethnicity included [36]. Several studies do not demonstrate differences in clinical CMA by race and ethnicity. A study that incorporated clinical and objective data in a New York City population from 1997 to 2007 found no differences in prevalence by race [52]. In another study, which compared prevalence based on self-report of reaction and physician diagnosis in a representative sample of the US population, non-Hispanic black and Asian participants had a statistically significant 50% lower risk of CMA than white participants, although limiting to those with a physician diagnosis decreased the precision and negated the significance of the estimate, and after restricting to those with positive OFC, no differences were seen by race [60]. It is unclear if disparities in access to care impacted these estimates. In the Wayne County Health, Environment, Allergy, and Asthma Longitudinal Study (WHEALS) birth cohort in Georgia, African American children were more likely to be sensitized based on sIgE to milk from birth through to age 3 to 6 years, but without increased clinically significant allergy [58]. 

There is evidence that genetic, epigenetic, and environmental factors play an important role in the development of CMA, although underlying mechanisms are still being elucidated [1,57]. CMA has been estimated to be 15% heritable [61], likely due to multiple genetic variants with small effect sizes. The prenatal and early childhood environment also plays a role, which is demonstrated by estimates of risk in immigrant populations. Among NHANES 2005–2006 participants aged 0 to 21 years, those who were US-born had a greater than 2-fold higher odds of sensitization to milk; among US-born children, those from immigrant families had a 1.7-fold higher risk of sensitization than children from non-immigrant households. There is evidence that children who immigrated in early life have a higher risk of sensitization to cow’s milk than those who immigrated later in life. Among immigrant children, those who immigrated prior to age 2 years had non-significantly increased odds of sensitization to cow’s milk (OR 3.47, *p* = 0.09) [62]. It will be important to further investigate the prenatal and early childhood exposures that underlie these differences and the implications on clinically relevant allergy.

## 10. Conclusions

Despite limitations in epidemiologic studies, the data support that CMA is an important problem worldwide with lifelong implications for health and, given its high prevalence in early life as well as natural history, may serve as a model for other food allergies. A better understanding of the epidemiology will guide clinician and public health efforts for prevention, diagnosis, and management of immediate hypersensitivity reactions, and treatment and prevention of growth restriction.

## Figures and Tables

**Table 1:** Methodological issues that affect prevalence estimates of Cow’s Milk Allergy (CMA).

Methodological Issue	
Variation in Reaction Types and Misclassification	- IgE-, mixed (IgE and non-IgE), and non-IgE mediated reactions - Non-immune mediated reactions (i.e., intolerance)
Study design	- Prospective cohort versus cross-sectional
Assessment of allergy	- Self-report - Physician diagnosis - Objective measures (sIgE, SPT) - Food challenge (DBPCFC—gold standard)
Diagnostic methods and distinguishing between sensitization and clinical allergy	- SPT and sIgE: Heterogeneity in method and assay used in study - Component-related diagnostics - Food challenge—double blind placebo controlled versus open
Study population	- High risk referral population versus average risk population based - Age at recruitment - Participation rates - Geographic region, genetic/environmental factors, differences in immigrant populations - Demographic factors (e.g., race/ethnicity, socioeconomic status)
Variations in phenotype	- Tolerate whole cow’s milk versus extensively heated baked milk - Variation in thresholds resulting in symptoms
Natural history	- Incomplete identification of resolved cases

**Table 2:** (A) Estimates of prevalence from meta-analyses and individual studies. **Meta-analyses.** (B) Estimates of prevalence from meta-analyses and individual studies. **Individual Studies.**

**(A)**			
**Authors**	**Methods**	**Prevalence of IgE-Mediated Food Allergy**	
Nwaru et al. [27]	Systematic review and meta-analysis of European studies published between 2000 and 2012 (includes 42 primary articles on CMA)	Self-report: 2.3% (95% CI 2.1%,2.5%) SPT alone: 0.3% (95% CI 0.03%,0.6%) sIgE alone: 4.7% (95% CI 4.2%,5.1%) Food challenge: 0.6% (95% CI 0.5%,0.8%) Positive Food challenge or history of reaction: 1.6% (95% CI 1.2%,1.9%).	
Rona et al. [3]	Meta-analysis of papers on food allergy published from January 1990 to December 2005	Prevalence Range Self-report: 1.2%–17% SPT alone: 0.2%–2.5% sIgE alone: 2%–9% Symptoms and sensitization: 0%–2.0% Food challenge: 0%–3.0%.	
**(B)**			
**Cohort**	***n*** **Age group**	**Prevalence of IgE-mediated CMA**	
EuroPrevall cohort [28]	*n* = 12049 enrolled *n* = 9336 followed to age 2Children	**Adjusted incidence:** 0.59% (adjusted for loss to follow-up/those not challenged/placebo reactors) **Natural History:** 57% developed tolerance within 1 year *Food allergy determined by (1) DBPCFC with sensitization on testing and no regular consumption of cow’s milk or (2) history of reaction or improvement with elimination*	
NHANES III (1988–1994) and NHANES 2005–2006 [36]	NHANES III *n* = 4995 NHANES 2005–2006 *n* = 2901 Children (age 6–19)	**Prevalence of sensitization to cow’s milk** **- Overall (sIgE ≥ 0.35):** NHANESIII: 8.3% (95% CI 7.0%,9.8%); NHANES 2005–2006: 8.1% (95% CI 6.1%,10.2%) **- Moderate-level (IgE ≥ 2 kU/L):** NHANESIII: 0.4% (95% CI 0.1%,0.7%); NHANES 2005–2006: 0.5% (95% CI 0.1%,0.9%) **- High-level (IgE ≥ 15, 95% predictive probability cut-off):** NHANESIII: 0%; NHANES 2005–2006: 0.008% (95% CI −0.01%,0.03%)	
NHANES 2005–2006 [32]	*n* = 8203 All ages	**Sensitization** Overall = 5.7% By Age 1–5 year = 22.0% 6–19 year = 8.1% 20–39 year = 3.2% 40–59 year = 4.9% ≥60 = 3.8%	**Estimated Clinical Food Allergy Rate** Overall = 0.40% By Age 1–5 year = 1.8% 6–19 year = 0.26% 20–39 year = 0.16% 40–59 year = 0.49% ≥60 = 0.33%
Melbourne, Australia High risk cohorts (first degree family history of atopy) [37]	Melbourne Atopy Cohort Study (MACS) (born 1990–1994), *n* = 620 High-risk subset of HealthNuts Study (born 2006–2010), *n* = 3661 Children	**Prevalence of sensitization (SPT)** MACS 2.4% (95% CI 1.6%,3.1%) HealthNuts 2.6% (95% CI 2.0%,3.4%)	
US Cross-sectional Telephone Survey of children (2009–2010) [7]	*n* = 38,380 Children	**Prevalence of self-reported CMA** 1.7% (95% CI = 1.5,1.8)	
US Internet/Telephone Survey of adults (2015–2016) [34]	*n* = 40,443 Adults	**Prevalence of self-reported CMA** 1.9% (95% CI = 1.8%,2.1%)	
NHANES 2007–2010 [33]	*n* = 20,686 All ages	**Prevalence of self-reported CMA** Children: 1.94% (95% CI = 1.43, 2.44) Adults: 2.64% (95% CI = 2.15, 3.13) *Prevalence in adults and children excluding those with ingestion = 1.62%* (95% CI = *1.32%, 1.92%*)	
New York City Urban Population (1997–2007) [52]	Retrospective chart review *n* = 9184 Median age 7 years, range 0–21 years	**Prevalence of physician documented CMA** = 0.5% *(0.3% excluding those with no specific symptoms and no confirmatory testing)*	
Israel, average-risk population (born 2004–2006) [29]	*n* = 13,019 Enrolled at birth and followed through age 3–5 year	**Cumulative incidence of CMA diagnosis** = 0.5%	
Denmark birth cohort (born 1985) [31]	*n* = 1749 Enrolled at birth	**Incidence Age 1 y** = 2.2% (95% CI = 1.5%,2.9%) **Incidence Age 1 y confirmed by OFC** = 0.5% (95% CI = 0.2%,0.9%)	
US Cross-sectional study of children (2009–2010) [60]	*n* = 3218	**Prevalence** = 1.6% (95% CI = 1.4%,1.7%)	

## Abbreviations

95% CI	95% Confidence Interval
CoFAR	Consortium for Food Allergy Research
CMA	Cow’s Milk Allergy
DBPCFC	Double Blind, Placebo-Controlled Oral Food Challenge
FPIES	Food Protein Induced Enterocolitis Syndrome
IgE	Immunoglobulin E
MACS	Melbourne Atopy Cohort Study
Mm	Millimeter
NHANES	National Health and Nutrition Examination Survey
OFC	Unblinded Oral Food Challenge
OR	Odds Ratio
sIgE	Serum-specific IgE (sIgE)
SPT	Skin Prick Test
US	United States
WHEALS	Wayne County Health, Environment, Allergy, and Asthma Longitudinal Study
